# Clinical Translation of Artificial Intelligence-Driven Gait Analysis Using Plantar Pressure and Ground Reaction Force

**DOI:** 10.3390/bioengineering13070796

**Published:** 2026-07-11

**Authors:** Junxiao Yang, Chunli Dong, Xiuping Zhang, Siyuan Tang, Haiya Sun

**Affiliations:** 1 School of Nursing, Jining Medical University, Jining 272000, China; 13721564399@163.com (J.Y.); 18653523209@163.com (C.D.); zhangxiuping816@163.com (X.Z.); 2 Xiangya School of Nursing, Central South University, Changsha 410013, China

**Keywords:** artificial intelligence, gait analysis, plantar pressure, ground reaction force, rehabilitation decision support

## Abstract

**Background**: Artificial intelligence (AI)-driven gait analysis using plantar pressure and ground reaction force (GRF) signals may provide objective digital biomarkers for rehabilitation, but clinical translation remains uncertain. This scoping review and evidence map aimed to summarize clinical applications, compare evidence maturity, and identify methodological and translational gaps. **Methods**: PubMed, Web of Science, Embase, and Scopus were searched from the earliest available indexed records in each database to May 2026. Original clinical studies using plantar pressure- or GRF-derived signals with AI methods for disease recognition, severity assessment, risk prediction, rehabilitation monitoring, or decision support were included. **Results**: Fifteen studies met the eligibility criteria. Evidence was concentrated in Parkinson’s disease (PD), particularly PD recognition and freezing of gait prediction, where relatively more mature evidence was supported by multiple studies and participant-level or cross-dataset validation. Evidence for PD severity assessment, knee osteoarthritis monitoring, chronic ankle instability rehabilitation, fall-risk stratification, sarcopenia screening, peripheral artery disease recognition, and functional gait disorder classification remained less mature. Translation was limited by small or single-center samples, unclear participant-level data splitting, limited external validation, absent calibration, sparse explainable AI reporting, and insufficient real-world workflow testing. **Conclusions**: Future studies should prioritize prospective, externally validated, interpretable, calibrated, and clinically embedded models before routine rehabilitation implementation.

## 1. Introduction

Gait disorders are commonly observed in neurological, musculoskeletal, vascular, metabolic, and geriatric diseases, serving as crucial clinical manifestations of impaired motor function and reduced independence [1]. During the rehabilitation process, gait assessment not only aids in determining the current functional status of patients but also provides a basis for efficacy observation and individualized intervention planning [2]. Although traditional observational gait assessments and clinical scales are widely used, their clinical utility may be limited by user dependence, episodic assessment, and difficulties in capturing subtle longitudinal changes [3,4]. For example, the assessment results are easily influenced by the assessors’ experience, and the information obtained mainly consists of fragmentary data at a certain time point, with limited ability to capture subtle and long-term changes in walking function. Therefore, there is still a clinical need for more objective, sensitive, and reproducible gait biomarkers to support precise rehabilitation assessment and the formulation of functional decisions [5,6].

Plantar pressure, center-of-pressure (CoP) trajectory, and ground reaction force (GRF) can directly reflect foot–ground interaction during walking. Specifically, plantar pressure assessment can quantify regional plantar loading, weight transfer, and pressure redistribution, providing clinically relevant information for foot-function evaluation and gait assessment [7,8]. GRF and CoP-derived variables are helpful for understanding weight-transfer patterns, postural-control strategies, shock absorption, and propulsion function during walking [9]. Currently, plantar pressure measurement systems and in-shoe pressure detection technologies have been widely used in foot function assessment, gait disorder identification, and portable monitoring [8,10]. GRF is also an important tool for quantifying human movement. However, due to its high data dimensionality, it is difficult to interpret the data manually [11]. In recent years, with the continuous development of wearable pressure insoles, pressure-sensing pads, and force sensor systems, the application of such signals has gradually expanded from laboratory evaluation to clinical follow-up and repeated monitoring in real life, which is particularly suitable for the continuous assessment of high-risk groups such as patients with diabetic foot [11,12].

Artificial intelligence (AI) methods are particularly suitable for analyzing plantar pressure and GRF data because these signals are high-dimensional, nonlinear, time-dependent, and affected by disease state, footwear, walking speed, sensor placement, and environmental conditions [13,14]. A recent systematic review on artificial neural network technology in plantar pressure analysis pointed out that multi-layer perceptrons, convolutional neural networks, and recurrent neural networks are among the most commonly used methods for plantar pressure-based modeling [15]. Moreover, in neural rehabilitation, machine learning (ML) has also been applied to gait analysis of diseases such as cerebral palsy and stroke. However, concerns still exist in aspects such as clinical relevance, feature selection, algorithm rationality, and real-environment verification [5]. Research on specific diseases further indicates that AI-based gait analysis has gone beyond simple disease identification. For example, vertical ground reaction force (vGRF) signals have been used to identify Parkinson’s disease (PD), and recently, force-sensor-based models have attempted to support the diagnosis and severity classification of PD [16]. ML models using instrumented walkway data have been developed for classifying multiple sclerosis, while wearable plantar pressure features have been used to detect radiological knee osteoarthritis (knee OA) and evaluate functional performance [6,17,18]. In patients with diabetic neuropathy and a history of foot ulcers, the combination of electromyography and GRF signals has also shown promise in multi-modal patient classification [19]. In acute ischemic stroke, ML based on CoP has been used to predict 3-month functional outcomes, indicating its potential value in rehabilitation-oriented prognosis prediction [20].

Disease-specific research further suggests that AI-driven gait analysis has evolved beyond simple disease recognition toward severity assessment, early warning, rehabilitation monitoring, and digital biomarker development. PD represents the most extensively studied application area because gait impairment is a core contributor to disability, reduced mobility, and diminished quality of life in affected individuals [21]. Within PD, freezing of gait (FOG) has emerged as a particularly important rehabilitation target because timely detection or prediction may facilitate cueing interventions and reduce mobility-related complications [22,23]. Beyond neurological disorders, gait-derived biomarkers have also attracted growing interest in musculoskeletal rehabilitation. Knee OA, one of the leading causes of mobility limitation worldwide, has been increasingly investigated using wearable gait-analysis technologies to support functional assessment and treatment monitoring [24]. Similar approaches have been explored in peripheral artery disease (PAD), where gait abnormalities may provide objective indicators of functional limitation and disease burden [25]. In aging populations, plantar pressure- and gait-derived features have also shown potential for sarcopenia screening and fall-risk stratification, both of which are highly relevant to preventive rehabilitation and long-term functional independence [26,27].

From a broader international and European perspective, sensor-based motion analysis and artificial intelligence-assisted decision support have also been applied in adjacent rehabilitation and engineering fields. A machine vision system based on Matlab is used to measure the upper limb movement trajectories during rehabilitation, supporting non-invasive measurement, visualization of rehabilitation progress, and electronic patient records [28]. Researchers explored the use of an inertial navigation system based on accelerometers and gyroscopes to achieve robot positioning, autonomous control, and tracking [29]. In industrial visual inspection, artificial intelligence object detection technology using transfer learning and an improved version of AlexNet architecture has achieved defect detection accuracy rates of 85.15% to 99.34% on different tire defect categories [30]. A diagnostic scheme for mechatronic systems based on neural networks was proposed, using high-performance data acquisition and processing sensors to obtain vibration signals and operating signals [31]. These studies demonstrate the common path from sensors to decisions, including data acquisition, preprocessing, interpretation based on artificial intelligence, and task-oriented decision support. This broader evidence further highlights the need to clearly define how the gait signals generated by foot pressure and ground reaction force (GRF) are transformed into AI applications with clinical relevance in the rehabilitation field.

Existing reviews have mainly summarized algorithmic approaches or disease-specific applications of machine-learning-based gait analysis. However, few reviews have specifically focused on foot–ground interaction signals, including plantar pressure, CoP, and GRF, while simultaneously comparing clinical tasks, validation robustness, external validation, interpretability, calibration, and implementation readiness. Therefore, this review addressed three questions: (1) in which disease populations and rehabilitation tasks have AI models using plantar pressure- or GRF-derived signals been evaluated? (2) how mature is the evidence when sample size, validation strategy, external validation, calibration, explainability, and real-world testing are considered? and (3) what methodological and translational gaps must be addressed before these models can support rehabilitation decision-making? The contribution of this review is to move beyond a descriptive algorithm summary and provide a structured evidence map and translational appraisal of AI-driven gait analysis based on foot–ground interaction signals. This review does not treat all clinical applications as a single homogeneous field, but instead compares evidence maturity across disease populations and rehabilitation tasks.

## 2. Methods

### 2.1. Research Design

This study was conducted through a systematic scoping review combined with an evidence map. This design was chosen because the aim was to describe the current clinical applications, evidence distribution, and translation gaps of AI-based gait analysis, rather than to conduct a pooled estimate of diagnostic or prognostic accuracy. Subsequently, the work was carried out in accordance with the established scoping review methodology and reported following the Preferred Reporting Items for Systematic Reviews and Meta-Analyses extension for Scoping Reviews (PRISMA-ScR) [32,33,34]. Reporting was additionally checked against the PRISMA 2020 checklist, and the completed checklist is provided as Supplementary Material. The article focused on using plantar pressure, GRF, or vGRF signals for AI-based gait analysis. The main clinical concerns included disease identification, severity assessment, risk prediction, rehabilitation evaluation, and wearable or home-based monitoring. Due to the expected differences among the included studies in terms of clinical populations, sensor systems, signal types, AI models, validation methods, and outcome definitions, a quantitative meta-analysis was not planned.

### 2.2. Search Strategy

A systematic literature search was conducted in PubMed, Web of Science, Embase, and Scopus from the earliest available indexed records in each database to May 2026, without applying a lower date restriction. Meanwhile, the reference lists of relevant studies and review articles were manually screened. The search keywords included gait analysis, plantar pressure, foot pressure, pressure insole, force plate, ground reaction force, vertical ground reaction force, center of pressure, machine learning, deep learning, artificial intelligence, diagnosis, severity, prediction, rehabilitation, and clinical decision support. The search strategies for each database were adjusted according to their characteristics. For the detailed search formula, please refer to the search strategy provided in Table S6 of the Supplementary Materials.

### 2.3. Inclusion and Exclusion Criteria

Inclusion Criteria

1. The study subjects are patients with various diseases presenting gait abnormalities, without restrictions on age, gender, and disease type.
2. The core analysis signals were plantar pressure or GRF data, without restrictions on the device type.
3. AI or ML algorithms were used for gait data analysis.
4. The outcome indicators involve disease identification, assessment of functional severity, or providing support for rehabilitation decision-making.
5. It is an original clinical study, and the full-text is accessible.

Exclusion Criteria

1. Plantar pressure or GRF was not used as the core analysis signal.
2. Only traditional biomechanical statistical methods were used, and no AI algorithms were applied.
3. Only pure biomechanical analysis is conducted, without involving relevant clinical applications.
4. Animal experiments and studies that only perform algorithm simulations without clinical data.
5. Studies with inaccessible full-text, duplicate publications, or non-English literature.

Several studies identified during background literature review were not included in the evidence synthesis because plantar pressure or GRF signals were not the primary analytical inputs, AI was not applied directly to plantar pressure/GRF gait modelling, or the studies did not meet the predefined eligibility criteria. Details are provided in Supplementary Table S1.

### 2.4. Research Screening and Data Extraction

All the retrieved records were imported into the reference management software EndNote 2025 and duplicate records were deleted. Two reviewers independently screened the titles and abstracts, and then evaluated the full texts of the potentially eligible studies. Disagreements were resolved through discussion, and in case of necessity, consultation with a third reviewer was sought. The screening process was summarized using the PRISMA flowchart [34].

A standardized data extraction form was used to collect information such as authors, publication year, study population, sample size, disease or functional status, sensor platform, signal type, gait task, AI model, validation strategy, clinical reference standards, primary performance indicators, and reported clinical applications. Studies related to PD and FOG were mainly classified as disease identification, severity assessment, and real-time risk prediction [35,36,37,38,39,40]. Musculoskeletal-related studies mainly included knee joint disease identification and rehabilitation assessment of chronic ankle instability [41,42]. Other clinical applications included sarcopenia screening, PAD classification, fall-risk assessment, functional gait disorder classification, and PD dual-task gait interpretation [43,44,45]. A total of 15 articles were ultimately included in the review.

### 2.5. Evidence Mapping and Methodological Assessment

For each included study, evidence mapping was conducted according to disease population, signal source, AI method, validation strategy, and intended clinical task. To describe clinical translation readiness, studies using laboratory force plates or public vGRF datasets were regarded as early-stage evidence, unless independent validation, cross-dataset testing, or clinical application was reported [46,47]. Studies using pressure insoles, shoe-integrated systems, real-time prediction models, or home-monitoring frameworks were considered closer to clinical translation, especially when the outputs were linked to monitoring, cueing, rehabilitation assessment, treatment follow-up, or clinical decision support [38,41,42,48].

Evidence maturity was classified descriptively using an a priori evidence-mapping matrix rather than by pooled effect estimation. Each clinical task was assessed across six domains: number of eligible studies, sample size and clinical diversity, validation strategy, external or cross-dataset validation, calibration and explainability reporting, and proximity to clinical implementation. “Emerging” evidence indicated single-study, laboratory-based, or dataset-based evidence with internal validation only. “Emerging–Moderate” evidence indicated evidence supported by at least one study with participant-level validation, independent device testing, cross-dataset validation, or clinically actionable outputs, but without prospective implementation. “Moderate” evidence indicated multiple studies using participant-independent or cross-dataset validation and a clearer rehabilitation action pathway, while still lacking sufficient evidence for routine clinical deployment. No application was rated as high-level evidence because prospective outcome validation, calibration, workflow integration, and real-world implementation testing were absent or limited across the included studies.

**Figure 1:**
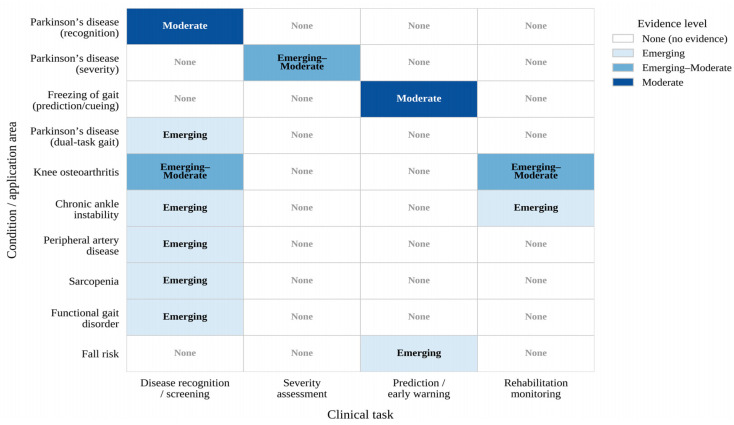
Evidence map of artificial intelligence (AI)-driven gait analysis using plantar pressure and ground reaction force. Evidence aggregation was performed at the disease population–clinical task level. Each cell summarizes the relative evidence maturity for a specific application area and task, based on the number of eligible studies, validation robustness, independent validation, calibration and explainability reporting, and proximity to clinical implementation. Evidence levels indicate relative maturity within the included evidence base, not definitive clinical effectiveness or readiness for routine implementation.

Because this review was designed as a scoping review, studies were not excluded solely because of methodological limitations. However, key methodological and translational issues were recorded, including sample size, participant-level validation, external validation, class imbalance, model interpretability, dataset overlap, calibration reporting, and real-world or wearable-device testing. For studies developing prediction models, methodological interpretation was informed by TRIPOD+AI (Transparent Reporting of a multivariable prediction model for Individual Prognosis Or Diagnosis plus Artificial Intelligence) and PROBAST+AI (Prediction model Risk Of Bias ASsessment Tool plus Artificial Intelligence) [49,50]. Particular attention was paid to whether repeated gait cycles, strides, or sliding windows from the same participant were appropriately separated during model training and testing. The evidence map presented in Figure 1 was generated from this evidence-mapping matrix. Rows represent disease populations or clinical application areas, columns represent clinical tasks, and each cell indicates the relative evidence maturity assigned according to the number of eligible studies, validation robustness, independent validation, calibration and explainability reporting, and proximity to clinical implementation.

### 2.6. Data Synthesis

The research findings were summarized in both textual and visual forms. Descriptive tables were employed to present the research characteristics, sensor platforms, signal types, AI models, validation methods, and clinical endpoints. An evidence map was also drawn to illustrate the distribution of various studies in terms of disease categories and clinical tasks.

Due to the significant differences among the included studies in aspects such as patient populations, signal acquisition systems, AI algorithms, validation procedures, reference standards, and outcome indicators, no pooled estimates were calculated. The comprehensive analysis focused on the clinical scenarios of the studies, the extent to which the applications advanced from simple classification to severity assessment or rehabilitation monitoring, and the obstacles still faced in the routine clinical implementation.

## 3. Results

### 3.1. Study Selection

**Figure 2:**
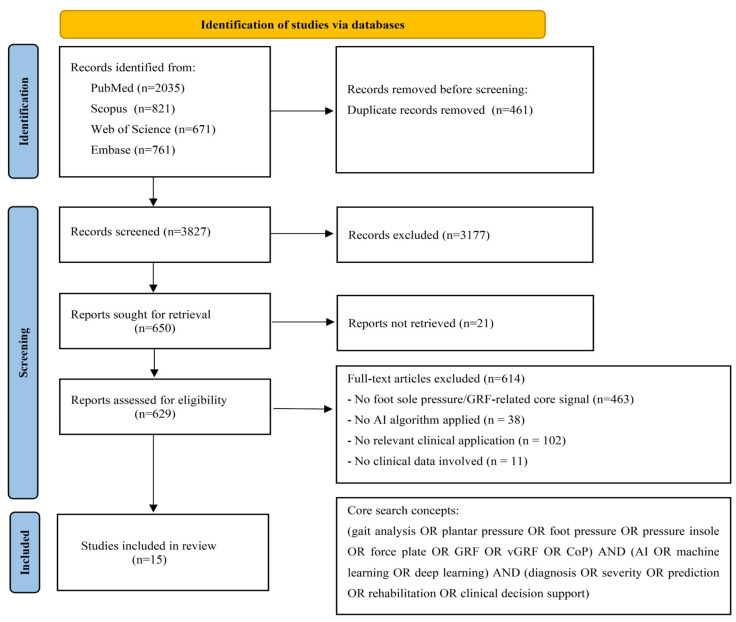
Preferred Reporting Items for Systematic Reviews and Meta-Analyses (PRISMA) flow diagram of study selection. GRF, ground reaction force; AI, artificial intelligence.

The process of study selection is presented in Figure 2. A total of 4288 records were identified from four databases, namely PubMed, Scopus, Web of Science, and Embase. After removing 461 duplicate articles, 3827 articles were screened based on their titles and abstracts, and 3177 irrelevant records were excluded. Among the 650 articles for which full-text was required, 21 could not be obtained. The eligibility for inclusion was evaluated for 629 full-text articles. Among them, 614 articles were excluded because they did not use plantar pressure or GRF as the core signals, did not apply AI algorithms, lacked relevant clinical applications, or did not involve clinical data. Finally, 15 studies were included in this systematic scoping review and evidence mapping.

### 3.2. Characteristics of the Included Studies

The main characteristics of the included studies are summarized in Table S2 of the Supplementary Materials. The included studies covered neurological, musculoskeletal, vascular, geriatric, and functional gait-related conditions. PD was the most frequently studied disease population, including studies on disease recognition, FOG prediction, dual-task gait analysis, and severity assessment. Other populations included knee OA or knee arthropathy, chronic ankle instability, PAD, sarcopenia, fall-risk populations, and functional gait disorders.

Signal acquisition methods varied across studies. GRF or vGRF signals were commonly used in studies of PD, functional gait disorders, knee OA, PAD, and dual-task gait analysis. Plantar pressure was collected using pressure insoles, in-shoe systems, pressure plates, or wearable plantar pressure devices. Several studies combined plantar pressure or GRF with inertial measurement unit (IMU) signals, CoP features, skeleton sequences, acceleration, angular velocity, or joint kinetic variables. These multimodal designs were mainly used for real-time prediction, severity assessment, rehabilitation monitoring, or clinical interpretability.

The included studies used a range of AI methods, including linear discriminant analysis (LDA), support vector machine (SVM), random forest (RF), extreme gradient boosting (XGBoost), decision-tree ensembles, convolutional neural networks (CNNs), long short-term memory (LSTM) networks, graph neural networks, and Transformer-based architectures. Traditional ML models were mainly applied to early-stage or small-scale clinical datasets, whereas deep-learning and hybrid models were more commonly used for time-series, multimodal, or wearable gait data.

**Figure 3:**
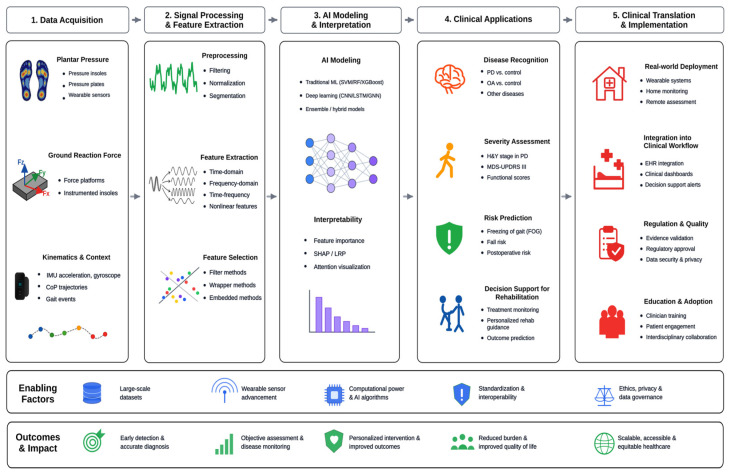
Clinical translation framework of plantar pressure- and ground reaction force (GRF)-based artificial intelligence (AI) in gait analysis. CoP, center of pressure; CNN, convolutional neural network; EHR, electronic health record; GRF, ground reaction force; H&Y, Hoehn and Yahr stage; IMU, inertial measurement unit; LRP, layer-wise relevance propagation; LSTM, long short-term memory; MDS-UPDRS III, Movement Disorder Society–Unified Parkinson’s Disease Rating Scale Part III; PD, Parkinson’s disease; RF, random forest; SHAP, Shapley additive explanations; SVM, support vector machine; XGBoost, extreme gradient boosting.

Overall, the included studies were concentrated in neurological applications, particularly PD and FOG. Evidence for musculoskeletal, vascular, geriatric, and functional gait applications was more limited. Figure 3 summarizes the clinical translation framework from signal acquisition to clinical application. In conceptual terms, the pathway can be expressed as: sensor-derived gait signals → feature extraction → AI modelling → clinical output → rehabilitation decision support. Specifically, plantar pressure, GRF/vGRF, CoP, and optional multimodal signals are first processed into gait features, including loading, temporal, symmetry, variability, and frequency-domain features. These features are then used by AI models to generate outputs such as disease recognition, FOG prediction, severity estimation, fall-risk screening, or rehabilitation-monitoring results. This expression clarifies the connection among the modules in Figure 3 without implying that all included studies used the same mathematical model.

### 3.3. Evidence Distribution Across Disease Populations and Clinical Tasks

The distribution of evidence across disease populations and clinical tasks is summarized in Table 1 and Figure 1, with study-level details provided in Supplementary Tables S3–S5. Evidence was unevenly distributed across clinical areas. Disease recognition/screening was the most frequently studied task, whereas severity assessment, risk prediction, rehabilitation monitoring, and decision-support applications were less frequently investigated. This uneven distribution appeared to correspond to differences in the amount and robustness of available evidence across clinical applications. As shown in Table 1, PD recognition and FOG prediction were supported by multiple studies and included internal, cross-dataset, participant-independent, or leave-one-freezer-out validation. In contrast, fall-risk stratification, sarcopenia screening, PAD recognition, and functional gait disorder classification were generally represented by single studies and were mainly supported by internal validation, with limited prospective outcome validation or external validation. Therefore, within the included evidence base, evidence maturity was relatively higher for PD recognition and FOG prediction, whereas broader rehabilitation decision-support applications remained at an emerging stage.

PD-related applications accounted for the largest number of included studies. For PD recognition, vGRF-based models and graph-based methods using public vGRF datasets were reported, and the evidence was rated as Moderate because both internal validation and cross-dataset validation were available [40,46]. PD severity assessment used vGRF, plantar pressure, CoP, and IMU signals to estimate Hoehn and Yahr (H&Y) stage or Movement Disorder Society-Unified Parkinson’s Disease Rating Scale Part III (MDS-UPDRS III) scores. Because the available studies were mainly single-center or internally validated, this application was rated as Emerging-Moderate [35,48].

FOG prediction was examined in four studies using plantar pressure alone or in combination with IMU signals [36,37,38,39]. These studies commonly used participant-independent or cross-dataset validation strategies. However, home-based performance and integration with cueing devices were not fully evaluated. Accordingly, the evidence for FOG prediction was rated as Moderate.

Musculoskeletal applications were represented by studies on knee OA and chronic ankle instability. One study used a platform-agnostic ML model based on vGRF features from force plates and digital insoles for knee OA recognition/monitoring [42]. Another study used a shoe-integrated plantar pressure and IMU system for chronic ankle instability recognition and postoperative rehabilitation assessment [41]. Knee OA monitoring was rated as Emerging-Moderate because independent digital-insole testing was reported, whereas chronic ankle instability remained Emerging because evidence was limited to a small internally validated cohort.

Other applications included PAD recognition, sarcopenia screening, functional gait disorder classification, and fall-risk stratification [43,44,45,47]. These applications were each supported by single studies with internal validation only and were therefore rated as Emerging. Overall, the evidence map showed that current research remains concentrated in recognition-oriented tasks, with fewer studies addressing longitudinal monitoring, rehabilitation decision support, or real-world implementation.

### 3.4. Model Performance and Validation Robustness

A summary of model performance and validation robustness is presented in Supplementary Table S4. Reported performance varied across clinical tasks and disease populations. For PD recognition, accuracy ranged from approximately 79.7% to 90.8%, with one study reporting sensitivity of 88.6% and specificity of 82.6% [40,46]. For FOG prediction, sensitivity ranged from 76.4% to 82.1%, and specificity ranged from 82.9% to 86.2% [36,37,39]. A subsequent study using an expanded dataset identified 86.8% of FOG episodes [38].

For PD severity assessment, one study reported MDS-UPDRS III prediction with an R^2^ of 0.87 and a root mean square error (RMSE) of 6.75, whereas another study reported H&Y severity classification accuracy of 98.8% [35,48]. For knee OA recognition/monitoring, the platform-agnostic model achieved an area under the receiver operating characteristic curve (auROC) of 0.86 using force-plate data and 0.83 using an independent digital-insole dataset [42]. For chronic ankle instability, the in-shoe model reported an accuracy of 93.4% and an area under the curve (AUC) of 0.959, although validation was limited to five-fold cross-validation (CV) [41].

Several validation and reporting limitations were identified. First, many studies did not clearly report whether data splitting was performed at the participant level. This is relevant because random splitting of repeated gait cycles, strides, or sliding windows from the same participant may overestimate model performance. Second, external validation was uncommon; only a small number of studies used cross-dataset validation, independent device testing, or independent datasets. Third, calibration was not reported in the included studies, limiting interpretation of predicted probabilities. Fourth, explainable artificial intelligence (XAI) methods were rarely reported, with only limited use of threshold-based interpretability or LRP. Finally, data and code availability were often unavailable or unclear. Therefore, reported performance should be interpreted together with validation robustness and reporting completeness.

### 3.5. Rehabilitation Decision Support and Clinical Translation Readiness

The rehabilitation decision support and clinical translation matrix is presented in Table S5 of the Supplementary Materials. FOG prediction/cueing models generated early-warning probabilities or binary FOG-onset predictions, with potential outputs linked to auditory, visual, or sensory cueing. The main implementation barriers were incomplete home-environment validation, possible false-positive cueing, and uncertain generalizability across FOG phenotypes.

PD severity monitoring models generated H&Y stage classifications or MDS-UPDRS III estimates from vGRF, plantar pressure, CoP, and IMU signals. The main reported barriers were lack of multicenter external validation, absence of longitudinal real-world monitoring, and no calibration reporting.

Musculoskeletal applications included knee OA functional monitoring and postoperative rehabilitation assessment after chronic ankle instability surgery. AI outputs included OA probability, digital gait biomarkers, pre-/postoperative gait changes, and classification of instability versus normal gait. These applications were limited by single-study evidence, small cohorts, limited longitudinal follow-up, and incomplete validation across daily functional tasks.

Other decision-support scenarios included fall-risk screening, PAD screening, sarcopenia screening, functional gait disorder classification, and PD dual-task gait interpretation. Across these applications, recurrent barriers included internal validation only, absence of prospective clinical endpoint validation, limited real-world testing, and lack of integration into routine rehabilitation workflows.

### 3.6. Summary of Evidence Gaps

Across the included studies, three recurrent evidence gaps were identified. First, several studies used small or single-center datasets, and participant-level splitting was often unclear. Second, external validation, calibration, XAI reporting, and data/code availability were limited. Third, few studies evaluated prospective outcomes, home-based performance, or integration into rehabilitation workflows. These gaps were observed across both neurological and non-neurological applications and are further interpreted in the Discussion.

## 4. Discussion

### 4.1. Principal Findings

This systematic scoping review mapped the clinical translation of AI-based gait analysis using plantar pressure, CoP trajectories, and GRF signals across 15 studies. Rather than showing uniform readiness across applications, the evidence suggests a task-specific maturity gradient, with some recognition- or prediction-oriented tasks being supported by relatively stronger validation evidence than rehabilitation-monitoring or decision-support tasks. The main finding is a mismatch between model development and clinical translation. Most studies reported classification or prediction performance, whereas fewer studies evaluated whether model outputs could support longitudinal monitoring, risk-threshold decisions, cueing, rehabilitation planning, or workflow integration. Therefore, the central issue is not whether AI models can achieve high performance in controlled datasets, but whether these outputs have been validated sufficiently to inform reproducible and actionable rehabilitation decisions. This pattern is consistent with previous reviews showing that AI-based gait analysis has expanded rapidly, while clinical relevance, validation quality, and real-world translation remain persistent concerns [5,15].

### 4.2. PD as the Dominant Application Area

PD was the dominant application area in the included evidence. This concentration is clinically understandable because gait impairment is common in PD and can be captured by foot–ground interaction signals. Earlier and recent studies have used vGRF and multimodal gait signals for PD recognition, severity classification, and clinical-scale estimation [16,35,46,48]. The key translational issue is whether these models can move beyond reproducing diagnostic or staging labels and provide information that is useful for monitoring progression, treatment response, and rehabilitation adjustment. At present, available evidence supports technical feasibility, but longitudinal validation against clinically meaningful outcomes remains limited. Thus, PD-related evidence can be considered relatively more mature within the included evidence base, but it should not be interpreted as sufficient evidence for routine rehabilitation implementation.

### 4.3. FOG as an Actionable Rehabilitation Target

FOG differs from general disease recognition because prediction can be linked directly to an intervention, such as auditory, visual, or sensory cueing. This makes FOG prediction a more action-linked task than cross-sectional disease classification, because the model output can theoretically trigger an immediate rehabilitation response. Plantar pressure modelling has demonstrated clinically relevant detection and prediction performance in PD [39]. However, the clinical threshold for a usable FOG alert is higher than model discrimination alone. A cueing system must operate reliably in daily environments, tolerate variable walking conditions, minimize false alarms, and remain acceptable to patients during repeated use. Therefore, the next translational step is not only improving accuracy but also testing real-time cueing systems prospectively in home and community settings.

### 4.4. Musculoskeletal Rehabilitation and Wearable Monitoring

Musculoskeletal applications were mainly represented by knee OA monitoring and chronic ankle instability rehabilitation assessment, both of which used wearable or force-based gait signals to support follow-up evaluation. The use of digital insoles and force-based gait features to monitor knee joint diseases or knee OA has been applied to remote assessment of functional status and treatment response [42]. Similarly, the in-shoe-integrated plantar pressure and IMU system for the monitoring of chronic ankle instability has shown how wearable sensing technology can support postoperative rehabilitation assessment and decision-making for resuming activities [41]. These studies are particularly relevant to rehabilitation practice as they link gait biomarkers to follow-up and rehabilitation monitoring, rather than just for one-time diagnosis. However, before these tools can be regarded as reliable rehabilitation monitoring systems, larger research cohorts, long-term follow-up, and validation in more diverse walking tasks (including stair climbing, turning, walking on uneven surfaces, and community activities) are required [52,53]. Such validation is especially important for rehabilitation applications because clinical decisions often depend on change over time rather than a single cross-sectional classification result.

### 4.5. Broader Clinical Applications and Evidence Gaps

Applications beyond PD and musculoskeletal rehabilitation broaden the possible clinical scope of plantar pressure- and GRF-based AI, but most remain at an early evidence stage. This is particularly important for fall-risk, geriatric, vascular, and functional gait applications, where clinical utility depends not only on cross-sectional classification but also on prospective prediction and actionable intervention pathways. A risk score is clinically meaningful only if it identifies future events with sufficient reliability and leads to changes in assessment, referral, prevention, or rehabilitation management.

### 4.6. Methodological Barriers to Clinical Translation

Several methodological barriers limit clinical translation. The first is data partitioning. Gait datasets often contain repeated strides, gait cycles, or sliding windows from the same participant. If these repeated samples are randomly split across training and test sets, performance may be overestimated because the model can learn participant-specific patterns rather than disease- or function-specific features. Participant-level splitting, leave-one-subject-out CV, and cross-dataset validation provide stronger evidence of generalizability. Without these strategies, apparently high model performance may partly reflect data leakage or participant-specific signal recognition rather than clinically generalizable gait patterns.

The second barrier is insufficient external validation. Wearable gait signals are sensitive to sensor placement, sampling frequency, footwear, walking speed, and environmental context. Without independent validation across devices, centers, and populations, it remains uncertain whether model performance can be maintained outside the original study setting.

The third barrier is calibration. Most included studies reported discrimination metrics such as accuracy, sensitivity, specificity, AUC, or F1 score, but calibration was generally absent. This matters because rehabilitation decisions often depend on risk or probability estimates rather than binary labels alone. Reporting frameworks for AI-based prediction models emphasize transparent reporting of predictors, outcomes, model development, validation, calibration, and intended clinical use [49,50].

The fourth barrier is interpretability. Rehabilitation clinicians need to understand which gait features are abnormal and how model outputs should inform treatment decisions. Outputs that identify impaired propulsion, loading asymmetry, abnormal CoP progression, impaired weight transfer, or increased variability are more clinically useful than black-box disease labels alone. XAI approaches such as LRP may help connect model predictions to interpretable gait features [51].

### 4.7. From Algorithmic Performance to Rehabilitation Decision-Making

A central translational gap is that high model performance does not automatically indicate clinical utility. Clinical utility requires evidence that model outputs can change decisions, improve timing of interventions, reduce uncertainty, or contribute to measurable patient benefit. For a model to influence rehabilitation practice, the clinical problem, target population, reference standard, decision threshold, output format, user, and workflow position must be clearly defined. Clinical translation therefore requires more than algorithm accuracy; it also requires reliable validation, interpretable outputs, workflow integration, and evidence that model use can improve decisions or outcomes [54]. Future studies should therefore evaluate how AI-derived gait biomarkers are used by clinicians and patients, rather than reporting performance metrics alone. This requires a shift from model-centered evaluation to decision-centered and workflow-centered evaluation.

### 4.8. Strengths and Limitations

This systematic scoping review presents multiple strengths. First, the review focuses specifically on plantar pressure and GRF signals, which are directly associated with foot–ground interaction and hold significant clinical value in the field of gait rehabilitation. Second, this study organizes and classifies existing evidence according to clinical tasks rather than solely by algorithm types, which enables clear differentiation among five categories of research: disease identification, severity assessment, risk prediction, rehabilitation monitoring, and decision support. Third, this review discusses four key dimensions, namely validation robustness, interpretability, model calibration, and clinical transformation readiness, all of which are frequently overlooked in existing literature on AI-based gait analysis.

This review also acknowledges several limitations that require clarification: First, as a scoping review, this study aims to map and present existing evidence rather than estimating pooled effect sizes for diagnostic or prognostic accuracy, hence no meta-analysis was conducted. Included studies exhibit considerable heterogeneity across disease populations, signal acquisition systems, walking task protocols, AI models, validation methods, reference standards, and outcome indicators, which does not meet the prerequisites for pooled analysis. Second, given the limited number of included studies, the classification of evidence maturity is provided only as descriptive reference and does not constitute definitive conclusions. Third, although this review documents significant methodological issues identified in included studies, it did not exclude relevant studies solely on the grounds of insufficient validation or incomplete reporting. While this approach is reasonable for evidence mapping, it implies that some study conclusions are only hypothesis-generating and have not yet met the criteria for clinical application. Fourth, this review may be subject to publication bias: studies with poor model performance or failed validation are less likely to be published, and thus may have been omitted.

## 5. Conclusions

This systematic scoping review and evidence map indicates that AI-driven gait analysis using plantar pressure, CoP, and GRF signals is a promising but still early-stage translational field in rehabilitation. Relatively more mature evidence was observed for Parkinson’s disease recognition and freezing of gait prediction, mainly because these areas were supported by multiple studies and participant-level or cross-dataset validation in at least some studies. In contrast, evidence for PD severity assessment, knee osteoarthritis monitoring, chronic ankle instability rehabilitation, fall-risk stratification, sarcopenia screening, peripheral artery disease recognition, and functional gait disorder classification remains less developed, with most applications limited to single-center cohorts, internal validation, small samples, or laboratory-based settings.

The core barrier to clinical translation is not model discrimination alone, but insufficient validation robustness, unclear participant-level data splitting, limited external validation, absent calibration reporting, sparse interpretability, and lack of integration with routine rehabilitation workflows. Future research should shift from retrospective classification studies toward prospective, multicenter, externally validated, interpretable, calibrated, and clinically embedded models. Priority should be given to evaluating whether AI-derived gait biomarkers can meaningfully improve rehabilitation assessment, treatment monitoring, intervention adjustment, cueing delivery, clinical referral, return-to-activity decision-making, and patient functional outcomes.

## Figures and Tables

**Table 1:** Comparative evidence maturity and clinical translation readiness across clinical applications.

Clinical Application	Evidence Base/Setting	Validation Robustness	Independent Validation	Translation Status	Evidence Maturity
PD recognition/screening [40,46]	2 studies; mainly public or laboratory vGRF datasets	Internal validation and cross-dataset testing	Partial; cross-dataset evidence, but no prospective clinical validation	Screening-oriented	Moderate
PD severity assessment [35,48]	2 small clinical studies; single-center settings	Internal model testing	No multicenter external validation	Severity monitoring	Emerging–Moderate
FOG prediction/detection [36,37,38,39]	4 small laboratory PD studies; mainly wearable plantar pressure datasets	Participant-independent or leave-one-freezer-out validation in several studies	Partial; no home-based prospective validation	Cueing support	Moderate
Knee OA/knee arthropathy [42]	1 study; force-plate dataset plus independent digital-insole dataset	Cross-validation and independent hold-out testing	Independent device/dataset validation	Monitoring/digital endpoint development	Emerging–Moderate
Chronic ankle instability [41]	1 prospective single-center cohort with postoperative subgroup	Five-fold cross-validation and internal rehabilitation assessment	No external validation	Rehabilitation assessment	Emerging
Peripheral artery disease [43]	1 laboratory gait cohort	Internal ML validation	No external validation	Screening hypothesis	Emerging
Sarcopenia [44]	1 single-center multimodal gait dataset	Internal deep-learning evaluation	No external validation	Screening hypothesis	Emerging
Functional gait disorder [47]	1 large retrospective gait-lab database	Internal LDA-based classification	No external validation	Gait-lab classification support	Emerging
Fall risk [45]	1 wearable plantar pressure study with compatibility testing	Internal two-stage model evaluation	Partial; no prospective fall-outcome validation	Risk screening	Emerging
PD dual-task gait interpretation [51]	1 gait-deterioration/interpretability study	Internal CNN/LRP evaluation	No external validation	Interpretability support	Emerging

Note: Evidence maturity reflects relative maturity within the included evidence base, not definitive clinical effectiveness or readiness for routine implementation. PD, Parkinson’s disease; FOG, freezing of gait; OA, osteoarthritis; vGRF, vertical ground reaction force; ML, machine learning; LDA, linear discriminant analysis; CNN, convolutional neural network; LRP, layer-wise relevance propagation.

## Data Availability

The original contributions presented in this study are included in this article. Further inquiries can be directed to the corresponding authors.

## Supplementary Materials

The following supporting information can be downloaded at: https://www.mdpi.com/article/10.3390/bioengineering13070796/s1. Table S1. Studies identified during background review but not included in evidence synthesis; Table S2. Study characteristics and sensing platforms (*n* = 15); Table S3. Evidence map by disease, signal modality, and clinical task; Table S4. Model performance and validation robustness (*n* = 15); Table S5. Rehabilitation decision-support and clinical translation matrix; Table S6. Search strategy.

## Abbreviations

The following abbreviations are used in this manuscript:

AI	Artificial intelligence
AUC	Area under the curve
auROC	Area under the receiver operating characteristic curve
CNN	Convolutional neural network
CoP	Center of pressure
CV	Cross-validation
DL	Deep learning
FOG	Freezing of gait
GRF	Ground reaction force
H&Y	Hoehn and Yahr
IMU	Inertial measurement unit
LDA	Linear discriminant analysis
LRP	Layer-wise relevance propagation
LSTM	Long short-term memory
MDS-UPDRS III	Movement Disorder Society–Unified Parkinson’s Disease Rating Scale Part III
ML	Machine learning
OA	Osteoarthritis
PAD	Peripheral artery disease
PD	Parkinson’s disease
PRISMA	Preferred Reporting Items for Systematic Reviews and Meta-Analyses
PRISMA-ScR	Preferred Reporting Items for Systematic Reviews and Meta-Analyses extension for Scoping Reviews
PROBAST+AI	Prediction model Risk Of Bias ASsessment Tool plus Artificial Intelligence
RF	Random Forest
RMSE	Root mean square error
SVM	Support vector machine
TRIPOD+AI	Transparent Reporting of a multivariable prediction model for Individual Prognosis Or Diagnosis plus Artificial Intelligence
vGRF	Vertical ground reaction force
XAI	Explainable artificial intelligence
XGBoost	Extreme gradient boosting

## References

[1] Pirker W., Katzenschlager R. (2017). Gait disorders in adults and the elderly: A clinical guide. Wien. Klin. Wochenschr..

[2] Baker R. (2006). Gait analysis methods in rehabilitation. J. Neuroeng. Rehabil..

[3] Simon S.R. (2004). Quantification of human motion: Gait analysis-benefits and limitations to its application to clinical problems. J. Biomech..

[4] Cimolin V., Galli M. (2014). Summary measures for clinical gait analysis: A literature review. Gait Posture.

[5] Samadi Kohnehshahri F., Merlo A., Mazzoli D., Bò M.C., Stagni R. (2024). Machine learning applied to gait analysis data in cerebral palsy and stroke: A systematic review. Gait Posture.

[6] Xie J., Li S., Song Z., Shu L., Zeng Q., Huang G., Lin Y. (2024). Functional Monitoring of Patients With Knee Osteoarthritis Based on Multidimensional Wearable Plantar Pressure Features: Cross-Sectional Study. JMIR Aging.

[7] Orlin M.N., McPoil T.G. (2000). Plantar pressure assessment. Phys. Ther..

[8] Abdul Razak A.H., Zayegh A., Begg R.K., Wahab Y. (2012). Foot plantar pressure measurement system: A review. Sensors.

[9] Warmerdam E., Hausdorff J.M., Atrsaei A., Zhou Y., Mirelman A., Aminian K., Espay A.J., Hansen C., Evers L.J.W., Keller A. (2020). Long-term unsupervised mobility assessment in movement disorders. Lancet Neurol..

[10] Ramirez-Bautista J.A., Huerta-Ruelas J.A., Chaparro-Cardenas S.L., Hernandez-Zavala A. (2017). A Review in Detection and Monitoring Gait Disorders Using In-Shoe Plantar Measurement Systems. IEEE Rev. Biomed. Eng..

[11] Horsak B., Slijepcevic D., Raberger A.M., Schwab C., Worisch M., Zeppelzauer M. (2020). GaiTRec, a large-scale ground reaction force dataset of healthy and impaired gait. Sci. Data.

[12] Castro-Martins P., Marques A., Coelho L., Vaz M., Baptista J.S. (2024). In-shoe plantar pressure measurement technologies for the diabetic foot: A systematic review. Heliyon.

[13] Topol E.J. (2019). High-performance medicine: The convergence of human and artificial intelligence. Nat. Med..

[14] Esteva A., Robicquet A., Ramsundar B., Kuleshov V., DePristo M., Chou K., Cui C., Corrado G., Thrun S., Dean J. (2019). A guide to deep learning in healthcare. Nat. Med..

[15] Wang C., Evans K., Hartley D., Morrison S., Veidt M., Wang G. (2024). A systematic review of artificial neural network techniques for analysis of foot plantar pressure. Biocybern. Biomed. Eng..

[16] Navita, Mittal P., Sharma Y.K., Rai A.K., Simaiya S., Lilhore U.K., Kumar V. (2025). Gait-based Parkinson’s disease diagnosis and severity classification using force sensors and machine learning. Sci. Rep..

[17] Hu W., Combden O., Jiang X., Buragadda S., Newell C.J., Williams M.C., Critch A.L., Ploughman M. (2022). Machine learning classification of multiple sclerosis patients based on raw data from an instrumented walkway. Biomed. Eng. Online.

[18] Li G., Li S., Xie J., Zhang Z., Zou J., Yang C., He L., Zeng Q., Shu L., Huang G. (2024). Identifying changes in dynamic plantar pressure associated with radiological knee osteoarthritis based on machine learning and wearable devices. J. Neuroeng. Rehabil..

[19] Haque F., Reaz M.B.I., Chowdhury M.E.H., Ezeddin M., Kiranyaz S., Alhatou M., Ali S.H.M., Bakar A.A.A., Srivastava G. (2022). Machine Learning-Based Diabetic Neuropathy and Previous Foot Ulceration Patients Detection Using Electromyography and Ground Reaction Forces during Gait. Sensors.

[20] Jeon E.T., Lee S.H., Eun M.Y., Jung J.M. (2024). Center of Pressure- and Machine Learning-based Gait Score and Clinical Risk Factors for Predicting Functional Outcome in Acute Ischemic Stroke. Arch. Phys. Med. Rehabil..

[21] Mirelman A., Bonato P., Camicioli R., Ellis T.D., Giladi N., Hamilton J.L., Hass C.J., Hausdorff J.M., Pelosin E., Almeida Q.J. (2019). Gait impairments in Parkinson’s disease. Lancet Neurol..

[22] Nutt J.G., Bloem B.R., Giladi N., Hallett M., Horak F.B., Nieuwboer A. (2011). Freezing of gait: Moving forward on a mysterious clinical phenomenon. Lancet Neurol..

[23] Nonnekes J., Snijders A.H., Nutt J.G., Deuschl G., Giladi N., Bloem B.R. (2015). Freezing of gait: A practical approach to management. Lancet Neurol..

[24] Hunter D.J., Bierma-Zeinstra S. (2019). Osteoarthritis. Lancet.

[25] Gornik H.L., Aronow H.D., Goodney P.P., Arya S., Brewster L.P., Byrd L., Chandra V., Drachman D.E., Eaves J.M., Ehrman J.K. (2024). 2024 ACC/AHA/AACVPR/APMA/ABC/SCAI/SVM/SVN/SVS/SIR/VESS Guideline for the Management of Lower Extremity Peripheral Artery Disease: A Report of the American College of Cardiology/American Heart Association Joint Committee on Clinical Practice Guidelines. Circulation.

[26] Cruz-Jentoft A.J., Bahat G., Bauer J., Boirie Y., Bruyère O., Cederholm T., Cooper C., Landi F., Rolland Y., Sayer A.A. (2019). Sarcopenia: Revised European consensus on definition and diagnosis. Age Ageing.

[27] Montero-Odasso M., van der Velde N., Martin F.C., Petrovic M., Tan M.P., Ryg J., Aguilar-Navarro S., Alexander N.B., Becker C., Blain H. (2022). World guidelines for falls prevention and management for older adults: A global initiative. Age Ageing.

[28] Kuryło P., Pivarčiová E., Cyganiuk J., Frankovský P. (2019). Machine Vision System Measuring the Trajectory of Upper Limb Motion Applying the Matlab Software. Meas. Sci. Rev..

[29] Qazizada M.E., Pivarčiová E. (2016). Mobile robot controlling possibilities of inertial navigation system. Procedia Eng..

[30] Kuric I., Klarák J., Bulej V., Sága M., Kandera M., Hajdučík A., Tucki K. (2022). Approach to Automated Visual Inspection of Objects Based on Artificial Intelligence. Appl. Sci..

[31] Stepanov P., Nikitin Y., Březina T., Jabloński R. (2014). Diagnostics of Mechatronic Systems on the Basis of Neural Networks with High-Performance Data Collection. Mechatronics 2013.

[32] Munn Z., Peters M.D.J., Stern C., Tufanaru C., McArthur A., Aromataris E. (2018). Systematic review or scoping review? Guidance for authors when choosing between a systematic or scoping review approach. BMC Med. Res. Methodol..

[33] Peters M.D.J., Marnie C., Tricco A.C., Pollock D., Munn Z., Alexander L., McInerney P., Godfrey C.M., Khalil H. (2020). Updated methodological guidance for the conduct of scoping reviews. JBI Evid. Synth..

[34] Tricco A.C., Lillie E., Zarin W., O’Brien K.K., Colquhoun H., Levac D., Moher D., Peters M.D.J., Horsley T., Weeks L. (2018). PRISMA Extension for Scoping Reviews (PRISMA-ScR): Checklist and Explanation. Ann. Intern. Med..

[35] Ji M., Dong H., Guo L., Li W. (2025). Diagnosis and Severity Rating of Parkinson’s Disease Based on Multimodal Gait Signal Analysis with GLRT and ST-CNN-Transformer Networks. IEEE J. Transl. Eng. Health Med..

[36] Pardoel S., Shalin G., Nantel J., Lemaire E.D., Kofman J. (2021). Early Detection of Freezing of Gait during Walking Using Inertial Measurement Unit and Plantar Pressure Distribution Data. Sensors.

[37] Pardoel S., Nantel J., Kofman J., Lemaire E.D. (2022). Prediction of Freezing of Gait in Parkinson’s Disease Using Unilateral and Bilateral Plantar-Pressure Data. Front. Neurol..

[38] Pardoel S., AlAkhras A., Jafari E., Kofman J., Lemaire E.D., Nantel J. (2024). Real-Time Freezing of Gait Prediction and Detection in Parkinson’s Disease. Sensors.

[39] Shalin G., Pardoel S., Lemaire E.D., Nantel J., Kofman J. (2021). Prediction and detection of freezing of gait in Parkinson’s disease from plantar pressure data using long short-term memory neural-networks. J. Neuroeng. Rehabil..

[40] Wang X., Xu X., Zhao Z., Li F., Qi F., Liang S. (2026). VGRF Signal-Based Gait Analysis for Parkinson’s Disease Detection: A Multi-Scale Directed Graph Neural Network Approach. IEEE J. Biomed. Health Inform..

[41] Guo Z., Li Y., Wang Y., Liu H., Guo R., Ma J., Wu X., Jiang D., Ren T. (2025). Intelligent Diagnosis and Predictive Rehabilitation Assessment of Chronic Ankle Instability Using Shoe-Integrated Sensor System. IEEE Trans. Neural Syst. Rehabil. Eng..

[42] Wipperman M.F., Lin A.Z., Gayvert K.M., Lahner B., Somersan-Karakaya S., Wu X., Im J., Lee M., Koyani B., Setliff I. (2024). Digital wearable insole-based identification of knee arthropathies and gait signatures using machine learning. eLife.

[43] Al-Ramini A., Hassan M., Fallahtafti F., Takallou M.A., Rahman H., Qolomany B., Pipinos I.I., Alsaleem F., Myers S.A. (2022). Machine Learning-Based Peripheral Artery Disease Identification Using Laboratory-Based Gait Data. Sensors.

[44] Naseem M.T., Kim N.H., Seo H., Lee J., Chung C.M., Shin S., Lee C.S. (2024). Sarcopenia diagnosis using skeleton-based gait sequence and foot-pressure image datasets. Front. Public Health.

[45] Song Z., Ou J., Wu S., Shu L., Fu Q., Xu X. (2025). Wearable fall risk assessment by discriminating recessive weak foot individual. J. Neuroeng. Rehabil..

[46] Farashi S. (2021). Analysis of the stance phase of the gait cycle in Parkinson’s disease and its potency for Parkinson’s disease discrimination. J. Biomech..

[47] Slijepcevic D., Zeppelzauer M., Gorgas A.M., Schwab C., Schuller M., Baca A., Breiteneder C., Horsak B. (2018). Automatic Classification of Functional Gait Disorders. IEEE J. Biomed. Health Inform..

[48] Huang J., Wang X., Jiang J., Liu F., Yang M., Wang S., Ma X., Chen W.M. (2026). A Wearable “Lab-in-Shoe” Gait Analysis System for Routine Clinical Assessment of People With Parkinson’s Disease. IEEE Trans. Neural Syst. Rehabil. Eng..

[49] Collins G.S., Moons K.G.M., Dhiman P., Riley R.D., Beam A.L., Van Calster B., Ghassemi M., Liu X., Reitsma J.B., van Smeden M. (2024). TRIPOD+AI statement: Updated guidance for reporting clinical prediction models that use regression or machine learning methods. BMJ.

[50] Moons K.G.M., Damen J.A.A., Kaul T., Hooft L., Andaur Navarro C., Dhiman P., Beam A.L., Van Calster B., Celi L.A., Denaxas S. (2025). PROBAST+AI: An updated quality, risk of bias, and applicability assessment tool for prediction models using regression or artificial intelligence methods. BMJ.

[51] Alharthi A.S. (2024). Interpretable machine learning comprehensive human gait deterioration analysis. Front. Neuroinform..

[52] Shen W., Hou Z., Wheeler P.C., Fong D.T.P. (2025). Minimizing running load via gentle heel strike techniques: A gait modification study. J. Sport Rehabil..

[53] Xie B., Zhang J. (2026). Assessment of plantar pressure dynamics in firefighters carrying self-contained breathing apparatus: A computational approach to gait stability analysis. Comput. Methods Biomech. Biomed. Engin..

[54] Vasey B., Nagendran M., Campbell B., Clifton D.A., Collins G.S., Denaxas S., Denniston A.K., Faes L., Geerts B., Ibrahim M. (2022). Reporting guideline for the early stage clinical evaluation of decision support systems driven by artificial intelligence: DECIDE-AI. BMJ.

