# Supplementary material for: Clinical Translation of Artificial Intelligence-Driven Gait Analysis Using Plantar Pressure and Ground Reaction Force
Source: Bioengineering (Basel). 2026 Jul 11;13(7):796. doi: 10.3390/bioengineering13070796 (PMC13406023; doi:10.3390/bioengineering13070796)
Supplement: Supplementary file 1 [file bioengineering-13-00796-s001.zip › bioengineering-4420269-supplementary.pdf]

Table S1. Studies identified during background review but not included in evidence synthesis.

Study	Reason for exclusion
Hu et al., 2022 [17]	Instrumented walkway gait analysis; plantar pressure/GRF not primary signal
Li et al., 2024 [18]	Outcome prediction based on multimodal gait variables; plantar pressure/GRF not core feature
Xie et al., 2024 [6]	Focused on wearable gait assessment; AI model not primarily developed using plantar pressure/GRF
Haque et al., 2022 [19]	Multimodal EMG + GRF classification; plantar pressure/GRF not analyzed independently
Jeon et al., 2024 [20]	Prognostic modeling using CoP variables after stroke; not focused on plantar pressure/GRF AI gait classification
Navita et al., 2025 [16]	Narrative background evidence; did not meet final eligibility criteria

Note: AI, artificial intelligence; CoP, center of pressure; EMG, electromyography; GRF, ground reaction force. Reference numbers throughout the Supplementary Materials correspond to the reference list in the main manuscript.

Table S2. Study characteristics and sensing platforms (n=15).

ID	Author/year	Population	Signal/device	AI model	Clinical task	Validation	Main finding	Translation role
S01	Slijepcevic et al., 2018 [47]	Functional gait disorders (hip/knee/ankle/calcaneus) healthy controls	GRF / laboratory GRF measurement system / force platform	GRF parameterization, PCA representations, LDA/classification experiments	Disease identification	Classification experiments; imbalance/session effects discussed	Framework for gait disorder classification; accuracy not reported	Baseline automated classification for clinically relevant gait-disorder categories
S02	Farashi, 2021 [46]	PD vs healthy controls	vGRF dataset/force-sensing system	Time-domain stance-phase vGRF features with classification analysis	Disease identification	Classification analysis	Accuracy 90.8%, sensitivity 88.6%, specificity 82.6%	PD discrimination from vGRF
S03	Pardoel et al., 2021 [36]	PD with FOG	Plantar pressure + IMU / pressure insoles + accelerometer/gyroscope sensors	Boosted decision-tree ensembles; mRMR/Relief-F feature selection	Prediction / early warning	Participant-independent validation; held out participant style evaluation	Combined PP-IMU model: sensitivity 76.4%; specificity 86.2%; FOG windows sensitivity 93.4%	Enables real-time FOG cueing using shoe-integrated sensors
S04	Shalin et al., 2021 [39]	PD with FOG	Plantar pressure / FScan pressure-sensing insoles, 100 Hz	2-layer LSTM using 16 plantar-pressure features	Prediction / early warning	Leave-one-freezer-out cross-validation; non-freezer specificity testing	Detection: sensitivity 82.1%; specificity 89.5%; prediction:	Real-time FOG detection/predictio n; Suitable for low-power,

							sensitivity 72.5%; specificity 81.2%; detected 95% freeze episodes	real-time FOG prediction
S05	Pardoel et al., 2022 [37]	PD with FOG	Plantar pressure / FScan pressure-sensing insoles, 100 Hz	RUSBoosted decision-tree ensembles; Relief-F feature selection	Prediction / early warning	Leave-one-freezer-out cross-validation	Bilateral model sensitivity 77.3%; specificity 82.9%; identified 94.2% FOG episodes 0.8 s before onset	Sensor-placement optimization for future FOG cueing systems
S06	Pardoel et al., 2024 [38]	PD with FOG	Plantar pressure / FScan pressure-sensing insoles, 100 Hz	Decision-tree ensemble classifiers across expanded datasets	Prediction / early warning	Comparison across three datasets; expanded training set	Dataset 3 model identified 86.84% total FOG episodes; 0.3 s earlier than Dataset 1 model	Authors describe readiness for implementation in a FOG prevention device
S07	Naseem et al., 2024 [44]	Sarcopenia vs non-sarcopenia	Foot-pressure image + skeleton sequence / foot-pressure plates + RGB-D skeleton capture	ResNet-18 for foot-pressure image; ST-GCN for skeleton sequence	Disease identification	4-fold cross-validation	Foot-pressure ResNet-18 accuracy 77.16%; skeleton ST-GCN accuracy 78.63%	Potential screening/diagnostic support for aging populations
S08	Wang et al., 2026 [40]	PD vs healthy controls across public vGRF datasets	vGRF / plantar sensor force time series / pressure-sensor-based vGRF datasets	MS-ADGNN with adaptive directed graph + multi-scale temporal convolution	Disease identification	Cross-dataset validation and 10-fold cross-validation	Cross-dataset ACC with augmentation: 79.69%, 86.51%, 80.36%; average accuracy improvement 2.46%	Objective diagnosis/treatment-effect monitoring; cross-dataset design improves reusability
S09	Song et al., 2025 [45]	Fall-risk / gait-disorder populations; compatibility tested on PD datasets	Wearable plantar pressure / wearable plantar-pressure system	Trainable threshold / two-stage individual-specific model	Risk prediction / stratification	Feasibility, ablation, enhanced datasets, compatibility on PD datasets	Two-stage model accuracy 85.4%; sensitivity 87.5%; individual-specific accuracies 87.5% and 73.6%	Quality control and individualized fall-risk screening
S10	Huang et al., 2026 [48]	PD across H&Y stages + healthy controls	Plantar pressure + CoP + IMU / Lab-in-Shoe with high-density pressure sensors and IMUs	ZUPT/PCA gait reconstruction; multiple linear regression for MDS-UPDRS III	Severity assessment	Gold-standard motion capture validation + clinical scale correlation	Stride length ICC 0.970, MAE 0.04 m; swing phase ICC 0.934; MDS-UPDRS III prediction R^2=0.87, RMSE=6.75	Supports clinical staging and treatment monitoring in PD
S11	Ji et al., 2025	PD severity classes +	vGRF + foot acceleration +	GLRT gait	Severity	Model evaluation	Classification	Automated PD

	[35]	healthy controls	angular velocity / multimodal wearable gait signal system	segmentation + ST-CNN-Transformer	assessment	on collected dataset	accuracy 98.81%	severity classification for clinical decision-making
S12	Wipperman et al., 2024 [42]	Knee arthropathy / knee OA vs controls	vGRF from force plate and digital insole / force plates + wearable digital insoles	Platform-agnostic ML model trained on force-plate data and applied to digital insole data	Disease identification / monitoring	Force-plate validation set + independent digital insole dataset	Force-plate validation auROC 0.86, auPR 0.90; independent digital-insole auROC 0.83, auPR 0.86	Supports at-home gait assessment and digital biomarker development
S13	Guo et al., 2025 [41]	Chronic ankle instability vs healthy; postoperative rehabilitation subset	Plantar pressure + IMU / shoe-integrated sensor system with graphene-based FSR insole sensors	XGBoost, compared with SVM/RF; t-SNE visualization	Disease identification / rehabilitation assessment	Five-fold cross-validation; pre/postoperative validation against medical records	Accuracy 93.39%; sensitivity 93.75%; specificity 92.68%; AUC 0.959	Post-op rehab assessment using plantar pressure + IMU
S14	Alharthi, 2024 [51]	PD and healthy dual-task gait/cognitive-load conditions	GRF / 16 underfoot force sensors; 100 Hz; public datasets	CNN with LRP	Disease identification / interpretability	Dataset-based classification; combined dataset analysis	F1 score 98% for PD dataset; 95.5% for combined PD dataset; 90% ± 10% for cognitive-load verification	XAI-based interpretation of gait deterioration patterns
S15	Al-Ramini et al., 2022 [43]	PAD vs healthy controls	GRF + joint angles/torques/powers / laboratory overground gait biomechanics system	Neural networks and RF algorithms	Disease identification	ML classification using all gait variables and GRF-only variables	All variables accuracy 89%; GRF-only accuracy up to 87%; MCC 0.64	Early PAD risk identification from gait signatures

Note: Abbreviations: ACC, accuracy; AI, artificial intelligence; AUC, area under the curve; auPR, area under the precision–recall curve; auROC, area under the receiver operating characteristic curve; CNN, convolutional neural network; CoP, center of pressure; FOG, freezing of gait; FSR, force-sensitive resistor; GLRT, generalized likelihood ratio test; GRF, ground reaction force; H&Y, Hoehn and Yahr; ICC, intraclass correlation coefficient; IMU, inertial measurement unit; LDA, linear discriminant analysis; LRP, layer-wise relevance propagation; LSTM, long short-term memory; MAE, mean absolute error; MCC, Matthews correlation coefficient; MDS-UPDRS III, Movement Disorder Society Unified Parkinson's Disease Rating Scale Part III; ML, machine learning; mRMR, minimum redundancy maximum relevance; MS-ADGNN, multi-scale adaptive directed graph neural network; OA, osteoarthritis; PAD, peripheral artery disease; PCA, principal component analysis; PD, Parkinson's disease; PP, plantar pressure; RF, random forest; RGB-D, red–green–blue depth; RMSE, root mean square error; ROC-AUC, receiver operating characteristic area under the curve; RUSBoost, random undersampling boost; ST-CNN, spatiotemporal convolutional neural network; ST-GCN, spatial–temporal graph convolutional network; SVM, support vector machine; t-SNE, t-distributed stochastic neighbor embedding; vGRF, vertical ground reaction force; XAI, explainable artificial intelligence; XGBoost, extreme gradient boosting; ZUPT, zero-velocity update.

Table S3. Evidence map by disease, signal modality, and clinical task.

Disease population	No. of studies	Signal modality	AI task	Main algorithms	Clinical endpoint	Validation level	Evidence maturity
PD recognition	2 (S02, S08)	vGRF	Recognition	Time-domain features, MS-ADGNN	PD vs control	Internal; cross-dataset	Moderate
PD severity	2 (S10, S11)	vGRF, CoP, IMU	Severity assessment	ST-CNN-Transformer, linear regression	H&Y stage, MDS-UPDRS III	Internal (single center)	Emerging–Moderate
PD with FOG	4 (S03, S04, S05, S06)	Plantar pressure + IMU	Prediction / early warning	Decision trees, LSTM, ensemble	FOG episode detection	Participant-level cross-validation	Moderate
PD + cognitive load	1 (S14)	GRF	Recognition / interpretability	CNN + LRP	PD under dual-task	Internal	Emerging
Knee OA / arthropathy	1 (S12)	vGRF (insole, force plate)	Recognition / monitoring	Platform-agnostic ML	OA diagnosis, digital biomarker	Internal + independent insole dataset	Emerging–Moderate
Chronic ankle instability	1 (S13)	Plantar pressure + IMU	Recognition / rehab assessment	XGBoost	Pre-/post-op gait change	Internal (5-fold CV)	Emerging
PAD	1 (S15)	GRF + joint kinetics	Recognition	Neural network, Random Forest	PAD vs control	Internal	Emerging
Sarcopenia	1 (S07)	Foot-pressure image	Recognition	ResNet-18, ST-GCN	Sarcopenia vs non-sarcopenia	Internal (4-fold CV)	Emerging
Functional gait disorders	1 (S01)	GRF	Recognition	PCA + LDA	Gait disorder type	Internal	Emerging
Fall risk	1 (S09)	Wearable plantar pressure	Risk stratification	Two-stage model	Fall risk score	Internal (enhanced datasets)	Emerging

Note: Evidence maturity is classified into three grades: Emerging, Emerging-Moderate, Moderate. Emerging evidence refers to evidence from single-center studies, laboratory-based research or internal validation; emerging-moderate evidence indicates that a certain degree of independent validation has been conducted, such as testing across multiple datasets or different devices, but the sample size is

limited or the study design remains single-center; moderate evidence indicates that validation at the subject level has been completed and/or multiple relevant studies have been conducted, although its application in real-world settings remains relatively limited. AI, artificial intelligence; CoP, center of pressure; FOG, freezing of gait; GRF, ground reaction force; H&Y, Hoehn and Yahr; IMU, inertial measurement unit; MDS-UPDRS III, Movement Disorder Society–Unified Parkinson's Disease Rating Scale Part III; OA, osteoarthritis; PAD, peripheral artery disease; PD, Parkinson's disease; vGRF, vertical ground reaction force

Table S4. Model performance and validation robustness (n=15).

Study	Clinical task	Reference standard	Data split strategy	Subject-level validation	External validation	Main performance metrics	Calibration reported	XAI reported	Data/code available
S01: Slijepcevic 2018 [47]	Functional gait disorder recognition	Clinical diagnosis	Not explicitly reported	Unclear	No	Accuracy not reported	No	No	No
S02: Farashi 2021 [46]	PD recognition	Clinical diagnosis	Not reported (likely cycle-level)	Unclear (likely No)	No	Acc 90.8%, Sen 88.6%, Spe 82.6%	No	No	No
S03: Pardoel 2021 [36]	FOG prediction	FOG annotation	Participant-independent	Yes	No	Sens 76.4%, Spec 86.2%	No	No	No
S04: Shalin 2021 [39]	FOG prediction	FOG annotation	Leave-one-freezer-out	Yes	No	Detection Sens 82.1%, 95% episodes	No	No	No
S05: Pardoel 2022 [37]	FOG prediction	FOG annotation	Leave-one-freezer-out	Yes	No	Sens 77.3%, Spec 82.9%	No	No	No
S06: Pardoel 2024 [38]	FOG prediction	FOG annotation	Cross-dataset (three datasets)	Yes	Partial (across datasets)	86.8% FOG episodes identified	No	No	No
S07: Naseem 2024 [44]	Sarcopenia recognition	Clinical diagnosis	4-fold cross-validation	Yes (implied by CV)	No	Acc 77.2% (foot image)	No	No	Unclear
S08: Wang 2026 [40]	PD recognition	Clinical diagnosis	Cross-dataset + 10-fold CV	Yes	Yes	Acc 79.7–86.5%	No	No	Unclear
S09: Song 2025 [45]	Fall risk stratification	Not specified	Enhanced datasets + compatibility test	Unclear	No	Acc 85.4%, Sen 87.5%	No	Yes (threshold interpretability)	Unclear
S10: Huang 2026 [48]	PD severity (MDS-UPDRS)	H&Y, MDS-UPDRS III	Training-testing split (implied)	Unclear	No	R^2=0.87, RMSE=6.75	No	No	Unclear
S11: Ji 2025 [35]	PD severity (H&Y)	Clinical	Not explicitly	Unclear	No	Acc 98.8%	No	No	Unclear

Study	Clinical task	Reference standard	Data split strategy	Subject-level validation	External validation	Main performance metrics	Calibration reported	XAI reported	Data/code available
		H&Y stage	reported						
S12: Wipperman 2024 [42]	Knee OA recognition / monitoring	Clinical OA diagnosis	Force-plate training, insole testing	Yes	Yes	auROC 0.86 (plate), 0.83 (insole)	No	No	Unclear
S13: Guo 2025 [41]	Ankle instability recognition / rehab	Medical records	5-fold cross-validation	Yes	No	Acc 93.4%, AUC 0.959	No	No	Unclear
S14: Alharthi 2024 [51]	PD recognition (dual-task)	Clinical diagnosis	Dataset-based split	Unclear	No	F1 98%	No	Yes (LRP)	Unclear
S15: Al-Ramini 2022 [43]	PAD recognition	Clinical diagnosis	Not explicitly reported	Unclear	No	Acc 89% (all variables), 87% (GRF only)	No	No	Unclear

Note: XAI, Explainable Artificial Intelligence; LRP, Layer-wise Relevance Propagation; CV, Cross-Validation; PD, Parkinson's Disease; FOG, Freezing of Gait; OA, Osteoarthritis; PAD, Peripheral Artery Disease.

Table S5. Rehabilitation decision-support and clinical translation matrix.

Rehabilitation domain	Clinical question	Input signal	AI output	Actionable rehabilitation implication	Current evidence level	Main barriers to implementation
FOG prediction/cueing	Can FOG episodes be predicted a few seconds before onset to trigger real-time cueing?	Plantar pressure + IMU (in-shoe)	Early-warning probability (0-1); binary FOG onset prediction	Activate auditory, visual, or sensory cueing device to prevent FOG; adjust walking strategy	Moderate (S03, S04, S05, S06; participant-level validation, but small samples, similar cohorts)	Real-time performance in home environment not fully validated; false positives may disturb patients; generalizability across different FOG phenotypes unknown
PD severity monitoring	Can AI estimate Hoehn & Yahr stage or MDS-UPDRS III score from gait signals, enabling continuous remote monitoring?	vGRF, plantar pressure, CoP, IMU	Severity class (H&Y 1-5) or continuous UPDRS score	Track disease progression over time; adjust medication or rehabilitation intensity; provide objective endpoint for clinical trials	Emerging–Moderate (S10, S11; S10 validated with motion capture, S11 single-center only)	Lack of external validation across multiple centers; no longitudinal validation with real-world data; calibration not reported
Fall risk	Can wearable plantar	Wearable plantar	Risk score	Recommend fall prevention	Emerging (S09; single study,	No prospective fall outcome

Rehabilitation domain	Clinical question	Input signal	AI output	Actionable rehabilitation implication	Current evidence level	Main barriers to implementation
screening in elderly	pressure data identify individuals with high fall risk, enabling home-based screening?	pressure	(continuous) or binary high-risk flag	training; home safety modifications; refer to physical therapy	internal validation only)	validation; threshold generalizability across different populations not tested
Knee OA functional monitoring	Can digital insoles estimate walking performance (e.g., gait speed, TUG) and detect OA status for home monitoring?	vGRF from force plate and insole	OA probability; digital gait biomarkers (e.g., symmetry, loading rate)	Adjust exercise prescription; evaluate response to orthotics or medication; remote follow-up	Emerging-Moderate (S12; independent insole dataset validation, but only regular walking)	Lack of longitudinal response data; not validated for other functional tasks (stairs, turning)
Post-operative rehabilitation after ankle surgery	Is recovery progressing objectively after chronic ankle instability surgery, and can remote guidance be provided?	Plantar pressure + IMU (shoe-integrated)	Pre-/post-operative gait change; classification of instability vs normal	Guide return-to-activity decisions; adjust rehabilitation protocol remotely; reduce in-person visits	Emerging (S13; small sample, 5-fold CV, validation against medical records)	Very small sample (single center); need larger cohorts and integration into clinical workflow
PAD screening	Can gait GRF patterns identify PAD for early referral and vascular assessment?	GRF + joint kinetics	PAD vs control classification	Prompt vascular laboratory referral; initiate supervised exercise therapy	Emerging (S15; single study, laboratory-based, no external validation)	No validation in primary care setting; no severity or progression monitoring
Sarcopenia screening in aging	Can foot-pressure images and skeleton sequences detect sarcopenia for early nutritional and exercise intervention?	Foot-pressure image + skeleton	Sarcopenia vs non-sarcopenia	Refer for dual-energy X-ray absorptiometry (DXA) confirmation; start resistance training and protein supplementation	Emerging (S07; moderate accuracy ~78%, 4-fold CV)	Low accuracy; not integrated with clinical workflow; no validation in real-world geriatric settings
Functional gait disorder	Can GRF patterns differentiate	GRF (force plate)	Classification into hip, knee, ankle,	Guide targeted physical therapy and psychological	Emerging (S01; framework only, quantitative accuracy not	No reported accuracy; purely laboratory-based; not validated for

Rehabilitation domain	Clinical question	Input signal	AI output	Actionable rehabilitation implication	Current evidence level	Main barriers to implementation
classification	functional gait disorders from healthy gait for appropriate psychiatric/physical therapy referral?		or calcaneus gait patterns	evaluation	reported)	clinical decision-making
PD dual-task gait deterioration interpretation	Can AI explain which gait features deteriorate under cognitive load, enabling individualized cognitive-motor training?	GRF	Feature importance maps (LRP) + classification	Design dual-task training focusing on the most affected gait parameters; monitor cognitive-motor interference	Emerging (S14; proof-of-concept, XAI demonstrated)	No clinical endpoint validation; small dataset; not tested as a feedback tool in rehabilitation

Note: Evidence level definitions: Emerging-single-center, laboratory-based, or internal validation only; Emerging-Moderate-some independent validation (e.g., cross-dataset, separate device) but small sample or single center; Moderate-participant-level validation, multiple studies, but still limited real-world deployment. PD, Parkinson's disease; FOG, freezing of gait; OA, osteoarthritis; PAD, peripheral artery disease; H&Y, Hoehn and Yahr; MDS-UPDRS III, Movement Disorder Society Unified Parkinson's Disease Rating Scale Part III; TUG, timed up-and-go; XAI, explainable artificial intelligence; LRP, layer-wise relevance propagation; CV, cross-validation.

Table S6. Search strategy

Database	Search Field	Search Strategy	Records
PubMed	Title/Abstract	("plantar pressure"[Title/Abstract] OR "foot pressure"[Title/Abstract] OR "ground reaction force"[Title/Abstract] OR GRF[Title/Abstract] OR "center of pressure"[Title/Abstract] OR COP[Title/Abstract] OR "pressure insole*"[Title/Abstract] OR "force plate*"[Title/Abstract]) AND ("artificial intelligence"[Title/Abstract] OR "machine learning"[Title/Abstract] OR "deep learning"[Title/Abstract] OR "neural network*"[Title/Abstract] OR "support vector machine*"[Title/Abstract] OR "random forest*"[Title/Abstract] OR classifier*[Title/Abstract] OR classification[Title/Abstract] OR prediction[Title/Abstract] OR predictive[Title/Abstract] OR algorithm*[Title/Abstract])	2035
Web of Science	Topic (title, abstract, keywords)	("plantar pressure" OR "foot pressure" OR "ground reaction force" OR GRF OR "center of pressure" OR COP OR "pressure insole*" OR "force plate*" OR "instrumented walkway*") AND ("artificial intelligence" OR "machine learning" OR "deep learning" OR "neural network*" OR algorithm* OR classifier* OR prediction model* OR "support vector machine" OR "random forest" OR CNN OR RNN)	671

Database	Search Field	Search Strategy	Records
		AND<br>(gait OR walking OR locomotion)<br>AND<br>(disease* OR diagnosis OR recognition OR classification OR severity OR "functional assessment" OR rehabilitation OR prognosis OR "outcome prediction" OR "decision support" OR monitoring)	
Scopus	TITLE-ABS-KEY	("plantar pressure" OR "foot pressure" OR "ground reaction force" OR GRF OR "center of pressure" OR COP OR "pressure insole*" OR "force plate*" OR "instrumented walkway*")<br>AND<br>("artificial intelligence" OR "machine learning" OR "deep learning" OR "neural network*" OR algorithm* OR classifier* OR prediction model* OR "support vector machine" OR "random forest" OR CNN OR RNN)<br>AND<br>(gait OR walking OR locomotion)<br>AND<br>(disease* OR diagnosis OR recognition OR classification OR severity OR "functional assessment" OR rehabilitation OR prognosis OR "outcome prediction" OR "decision support" OR monitoring)	821
Embase	Default (title, abstract, keywords)	("plantar pressure" OR "foot pressure" OR "ground reaction force" OR GRF OR "center of pressure" OR COP OR "pressure insole*" OR "force plate*" OR "instrumented walkway*")<br>AND<br>("artificial intelligence" OR "machine learning" OR "deep learning" OR "neural network*" OR algorithm* OR classifier* OR prediction model* OR "support vector machine" OR "random forest" OR CNN OR RNN)<br>AND<br>(gait OR walking OR locomotion)<br>AND<br>(disease* OR diagnosis OR recognition OR classification OR severity OR "functional assessment" OR rehabilitation OR prognosis OR "outcome prediction" OR "decision support" OR monitoring)	761

Note: The truncation symbol * is used for plural and suffix variants. All searches were performed from the earliest available indexed records in each database to May 2026, without applying a lower date restriction. The record counts are approximate and reflect the date when the search was executed; they may vary slightly over time.
